# Reliability and accuracy of intraoperative fluoroscopy assessment of acetabular cup anteversion in supine direct anterior approach total hip arthroplasty

**DOI:** 10.1038/s41598-024-62964-6

**Published:** 2024-05-30

**Authors:** Weihua Li, Yan Huang, Zehui Zou, Xuqiang Liu, Xiaofeng Li

**Affiliations:** 1 Orthopedic Hospital, The First Affiliated Hospital, Jiangxi Medical College, Nanchang University, Nanchang, China; 2 Artificial Joints Engineering and Technology Research Center of Jiangxi Province, The First Affiliated Hospital, Jiangxi Medical College, Nanchang University, Nanchang, China

**Keywords:** Total hip arthroplasty, Direct anterior approach, Intraoperative fluoroscopy, Cup anteversion, Trauma, Orthopaedics

## Abstract

Poor implantation positioning of hip prostheses is considered the primary factor affecting postoperative joint wear. Cup anteversion in direct anterior approach (DAA) total hip arthroplasty (THA) is often excessive. Intraoperative fluoroscopy (IF) are effective for improving implant placement accuracy. This study aimed to analyze IF’s reliability and accuracy in assessing intraoperative anteversion. Sixty-two consecutive hips underwent primary THA utilizing DAA alongside IF for cup placement. Intraoperative anteversion was measured using IF images, while postoperative CT and standard anteroposterior (AP) radiographs were used to calculate true anteversion component angles. Differences and correlations between intraoperative and true anteversions were analyzed, and intraclass correlation coefficients (ICC) determined the inter- and intra-observer reliabilities. Excellent intra- and inter-observer reliabilities were observed for all radiographic and CT methods (ICC > 0.9). Strong correlations (PCC > 0.6) existed between anteversion measured on IF image and postoperative CT and AP pelvic measurements. Intraoperative anteversion measured on IF images (16.8 ± 3.2°) was smaller than anteversion measured postoperatively on AP X-rays (21.3 ± 4.7°, *P* < 0.001) and CT (22.0 ± 4.9°, *P* < 0.001), with average differences of 4.5°and 5.3°, respectively. Under several influencing factors, the accuracy of IF in assessing cup anteversion in DAA-THA may be limited. However, this still requires large-sample experiments for verification.

## Introduction

Total hip arthroplasty (THA) is currently the best treatment of choice for advanced hip diseases, with direct anterior approach (DAA) being a popular surgical technique due to its advantages of reduced trauma, lower dislocation rates, and faster recovery compared to the posterolateral approach^1,2^. However, in DAA-THA, acetabular cup anteversion is often excessive due to femoral interference and the uneven force of the surgical equipment during implantation, which increases the long-term wear rate of the prosthesis^3,4^.

Improper acetabular component positioning is well known to be a major cause of impingement, dislocation, and wear after THA. Acetabular loosening, linear wear, and femoral loosening remain the top three reasons for revision following primary THA^5^. To improve acetabular cup orientation, researchers recommend the use of intraoperative fluoroscopy (IF) for DAA. Unlike computed tomography (CT)-based 3D navigation techniques, IF is easy to perform. However, some researchers argued that IF images were not reliable for improving implant positioning or sizing^1,2^. Stereometric effects (parallax) in 2D projections affect intraoperative angle measurements, which may not reflect the true angle of the intraoperative prosthesis to a certain extent^6,7^. IF measurements may differ from postoperative standardized plain radiographs and CT measurements.

In previous studies, the calculation of the acetabular cup anteversion based on postoperative anteroposterior (AP) pelvic radiographs had high accuracy. Among several measures, the "Lewinnek" method was widely used because of its extremely high accuracy and higher intra- and inter-observer reliabilities^8,9^. Nonetheless, the reliability and accuracy of IF for assessing intraoperative anteversion are currently unknown. In this study, we hypothesized that there were non-equivalent results between anteversion based on IF and postoperative measurements. This study measured intraoperative and postoperative acetabular cup anteversion using the Lewinnek method and the gold-standard CT method to assess the consistency between IF and standardized measurements.

## Materials and methods

### Patient recruitment

Patients who underwent DAA-THA between September 2021 and February 2022 were included in this study. Implantation with an uncemented acetabular component was performed in all included patients. The study protocol was approved by the Ethics Committee of the First Affiliated Hospital of Nanchang University, and all methods were performed in accordance with relevant guidelines and regulations, with written informed consent obtained from all participants. Exclusion criteria comprised: (1) patients undergoing hip revision surgery; (2) patients with ankylosing spondylitis; (3) patients who had undergone lumbosacral fusion; (4) patients with a severe spinal degenerative disease or pelvic tilt; (5) patients with congenital hip dysplasia (Crowe III-IV type); (6) patients with a history of a hip joint infection or osteotomy on the affected side; (7) patients with residual internal fixation in the hip joint and the acetabular prosthesis not clearly displayed on a CT scan; (8) patients with incomplete clinical data.

### Surgical approach (acetabular side)

Patients were positioned supine and epidural anesthesia was administered. After routine sterilization and draping, a surgical incision was positioned using the anterior superior iliac spine (ASIS) as a bony landmark, and the subcutaneous tissue and fascia were incised layer-by-layer. The superficial layer was entered along the gap between the tensor fascia lata and sartorius muscle, and the deep layer was entered along the gap between the rectus femoris and lateral femoris muscle. The "L" shape was used to cut the joint capsule and expose the femoral neck. The femoral neck was cut above the lesser trochanter and conventional THA instruments were used to ream the acetabulum and implant the joint prosthesis.

### Radiological method of calculating the anteversion

Patients underwent X-ray examinations, including pelvic AP and lateral hip imaging, following the removal of the drainage tube but prior to weight-bearing. The standard intraoperative and postoperative AP X-ray film method for the pelvis involved positioning the patients in a supine position with the lower limbs inwardly rotated by 10°–15°. The X-ray beam was focused perpendicular to the body and centered on the pubic symphysis at a height of 110 cm. To exclude excessive pelvic tilt effects on the measurement of acetabular cup anteversion, the vertical distance between the upper edge of the symphysis and sacrococcygeal joint midpoint was calculated from the postoperative AP pelvic plain film to standardize the images. As described by Tannast et al., the distance was considered "appropriate" if it was between 20–40 mm in male patients and 20–55 mm in female patients^10,11^. In this study, the mean distance was 33.1 mm in men (range; 27.6–37.3 mm) and 46.3 mm in women (range: 26.8–52.7 mm). Therefore, none of the patients included in this study had excessive pelvic tilt, which did not significantly affect the study results.

Fluoroscopy was performed to determine the position of the acetabular cup after placement. First, the C-arm machine was adjusted such that the midline of the sacrum was aligned with the pubic symphysis. The obturator shape on the fluoroscopic image was then matched to that on the preoperative supine AP pelvic x-ray. The final step was to center the pubic symphysis on the projection and save the fluoroscopic image. Postoperative CT scan for patients was performed after removal of the drainage tube and before discharge from the hospital. The scope of the bilateral hip CT scans included the entire pelvis up to 15 cm below the lesser trochanter of the femur, with a 1:1 pitch in transverse slices, spiral scanning, and an image resolution of 512 × 512.

**Figure 1:**
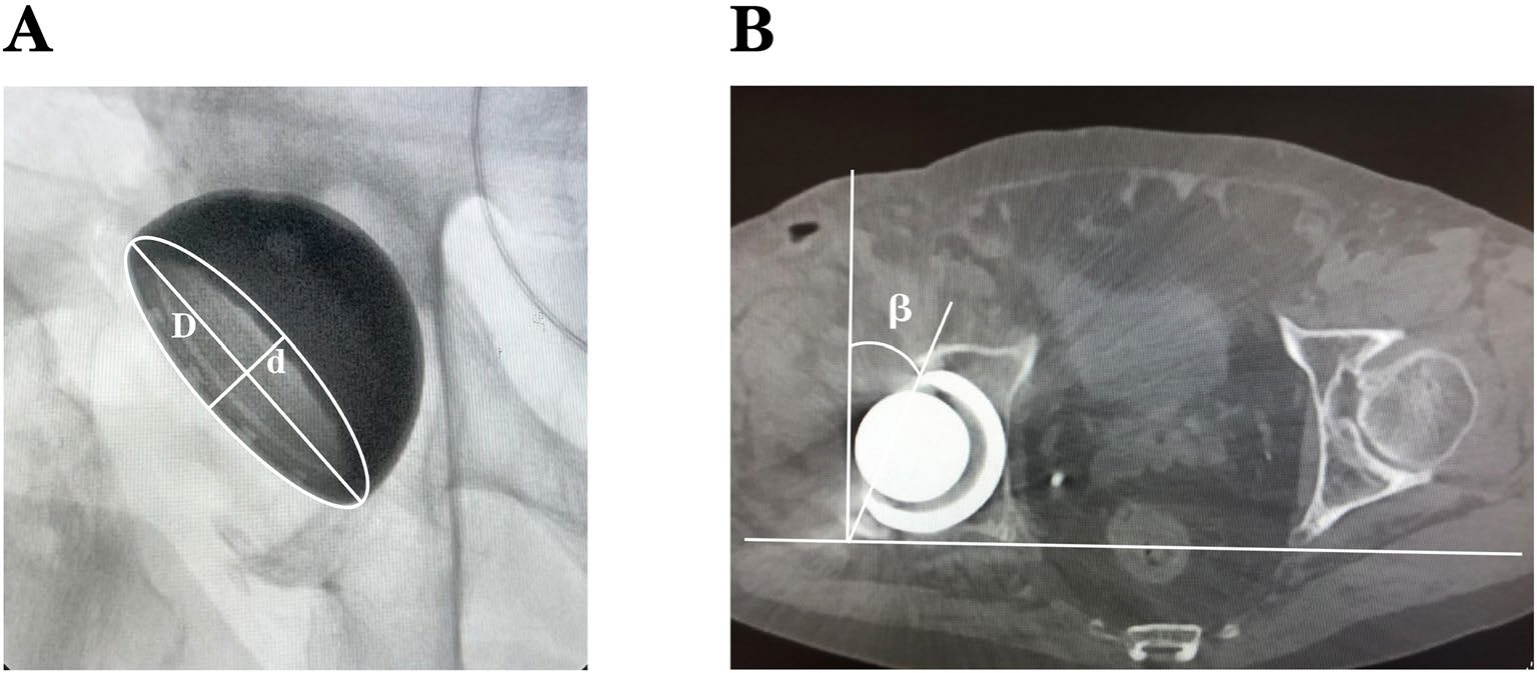
Measurement of the anteversion by the Lewinnek method (**A**) and CT scan (**B**). d: minor axis; D: major axis; β: anteversion.

Measurement of acetabular cup anteversion using the Lewinnek method. The obtained X-ray films were imported into ImageJ software. Using the software to mark the outermost edge of the acetabular cup and its minor and major axes, the minor axis was set to a unit length of 1 mm (Fig. 1A).

Lewinnek method:

$$\text{Anteversion }= {\text{sin}}^{-1}\left(\frac{d}{D}\right)$$

where d: minor axis D: major axis.

CT measured the anteversion as the angle (β) between a line connecting the border of the acetabular cup and a reference line drawn perpendicular to a line between the posterior pelvic margins at the level of the sciatic notch (Fig. 1B).

### Statistical analysis

Statistical Package for Social Sciences 26 (SPSS 26, Chicago, Illinois, USA) and ImageJ software (version 1.48) were used for data analysis. Calculations using the G*Power software (version 3.1) software yielded a minimum of 34 patients required for this study (parameters: effect size d = 0.8, power = 0.9, and alpha failure of α = 0.05). General demographic characteristics were presented as mean (SD) or n (%). The radiometric measurements were performed independently by three observers. The intraclass correlation coefficient (ICC) was used to evaluate the intra-observer and inter-observer reliability, with values ranging between 0.6 and 0.8 for medium repeatability, and 0.8 and 1 for high repeatability. The correlation between the different measurements was analyzed using the Pearson correlation coefficient (PCC), with values ranging from 0.8–1.0 for an extremely strong correlation, 0.6–0.8 for a strong correlation, 0.4–0.6 for a moderate correlation, and 0.2–0.4 for a poor correlation. Paired t-test was performed to determine the significance between quantitative variables, and differences were considered statistically significant at *P* < 0.05.

## Result

### Patient characteristics

Sixty-two patients were included in this study, comprising 23 males and 39 females, with a mean age of 62.2 (SD: 11.7) years and a body mass index (BMI) of 22.2 (SD: 2.6) kg/m^2^. ASA II and III classifications accounted for 38.7% and 61.3% of the cases, respectively. The preoperative diagnoses were osteoarthritis in 2 hips, developmental dysplasia of the hip (Crowe type I or II dysplasia) in 4 hips, femoral neck fracture (Garden types III and IV) in 14 hips, and osteonecrosis of the femoral head in 42 hips (Table 1).

**Table 1:** Clinical characteristics of patients (N = 62).

Parameters	Mean (SD)/n(%)
Age (years)	62.2 (11.7)
Gender (males)	23 (37.1%)
BMI (kg/m^2^)	22.2 (2.6)
ASA classification	
II	24 (38.7%)
III	38 (61.3%)
Preoperative diagnosis	
Severe osteoarthritis	2 (3.2%)
DDH	4 (6.5%)
Femoral neck fracture	14 (22.6%)
ONFH	42 (67.7%)

Values are n, n (%), or mean (SD); ASA, American society of anesthesiologists; SD, Standard deviation; BMI, Body mass index. DDH, Developmental dysplasia of the hip; ONFH, Osteonecrosis of the femoral head.

### Assessment of reliability and correlation

The interobserver and intraobserver ICCs of the three measurement methods exceeded 0.9, indicating high reliability (Table 2). The anteversion measurements by IF images, postoperative pelvic AP plain film, and postoperative CT scan were 16.8 ± 3.2°, 21.3 ± 4.7°, and 22.0 ± 4.9°, respectively (Table 3). Compared with the CT scan, no significant difference was observed in anteversion measured by postoperative AP pelvic radiographs (P = 0.328). However, the intraoperative anteversion measured using IF images was significantly smaller than the postoperative anteversion measured by AP X-rays and CT scans (*P* < 0.001). In addition, the PCC values exceeded 0.6, indicating a strong correlation between the different measurement methods.

**Table 2:** Intra-observer and inter-observer reliability of measuring method.

Method	Intra-observer reliability (ICC, 95% CI)	Inter-observer reliability (ICC, 95% CI)
Intraoperative fluoroscopy	0.940 (0.902–0.963)	0.951 (0.920–0.970)
Postoperative AP X-ray	0.961 (0.936–0.976)	0.969 (0.949–0.981)
Postoperative CT scan	0.957 (0.929–0.974)	0.971 (0.953–0.983)

ICC, Intraclass correlation coefficient; CI, Confidence interval; AP: Anteroposterior.

**Table 3:** Results and correlation analysis of different measurement methods.

Method	Anteversion angle	As compared to CT scan				As compared to postoperative AP X-ray		
		Mean difference	*P*-value	PCC		Mean difference	*P*-value	PCC
Postoperative CT scan	22.0 ± 4.9	Ref	Ref	Ref		–	–	–
Intraoperative fluoroscopy	16.8 ± 3.2	5.3	< 0.001*	0.661		4.5	< 0.001*	0.631
Postoperative AP X-ray	21.3 ± 4.7	0.7	0.328	0.959		Ref	Ref	Ref

PCC: Pearson correlation coefficient; AP: Anteroposterior; *: Statistically significant.

### Safe zone outliers and correction factor

**Figure 2:**
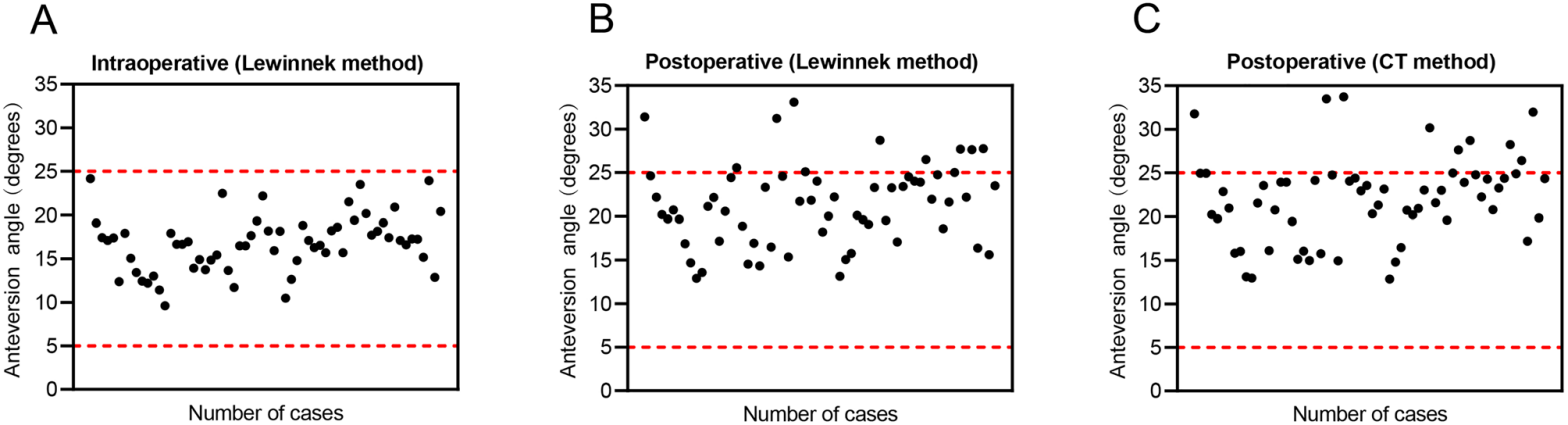
Scatter diagram of anteversion measured by different measurement methods. The inside of the red dotted line represented the range of the safe zone. (**A**) Intraoperative fluoroscopy; (**B**) Postoperative AP X-ray; (**C**) CT scan.

**Figure 3:**
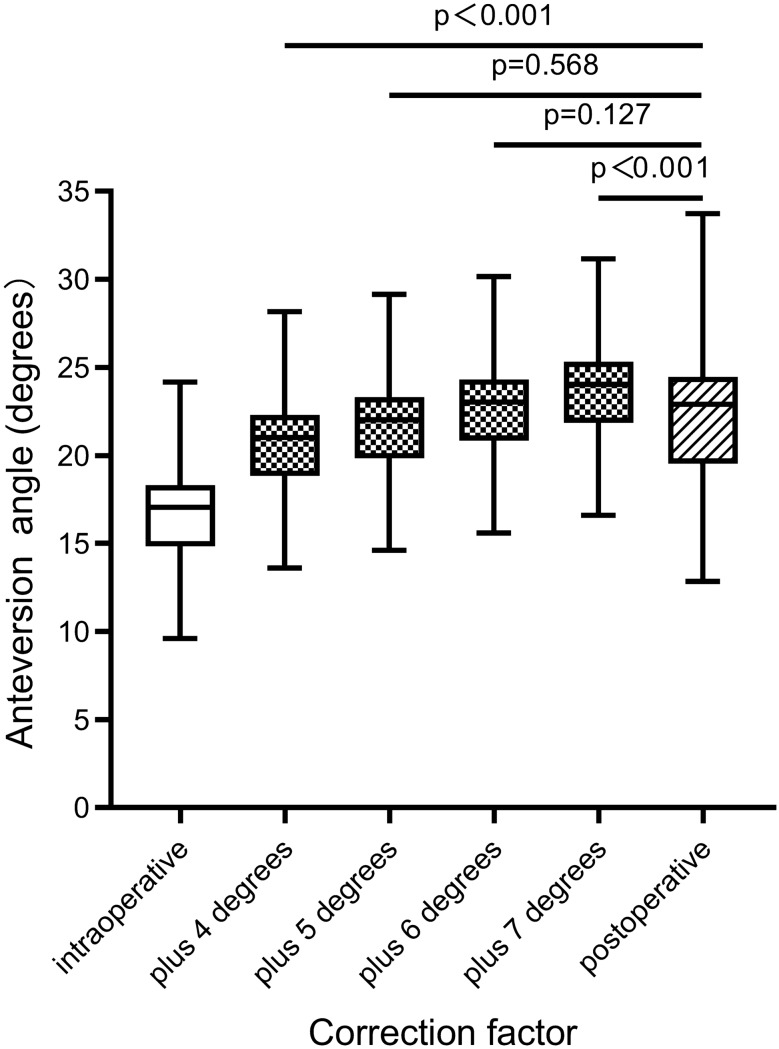
The difference analysis between intraoperative and postoperative anteversion after adding correction factors.

The intraoperative anteversions measured using IF images were all within the Lewinnek safety zone (Fig. 2A). The proportions of anteversion measured using postoperative pelvic AP radiographs and CT scans in the safe zone were 82.3% and 85.5%, respectively, with all outliers outside the safe zone having an anteversion greater than 25° (Fig. 2B, C). Figure 3 shows that adding a 5° or 6° correction factor to intraoperative anteversion did not significantly differ from the postoperative anteversion measured using postoperative CT scan.

## Discussion

Poor implantation positioning of hip prostheses is considered the leading factor affecting postoperative joint function and durability. Malpositioning of the acetabular component increases the risk of prosthetic impingement, wear, and dislocation after THA^12^. According to Lewinnek's definition of the "safe zone" for acetabular prosthesis positions, the abduction angle of the acetabular cup should be between 30° and 50° and the anteversion should be between 5° and 25°^13^. Outside this region, the acetabular cups are subjected to higher biomechanical stress, resulting in markedly increased rates of polyethylene wear and osteolysis^14^. Recently, researchers have questioned the so-called “safe zone” concept because it is not entirely safe^15,16^. However, this remains the primary target of contemporary THA.

Although computer navigation, surgical robotics, and intraoperative CT can assist in the precise positioning of components, their high costs and radiation exposure limit their widespread use. In contrast, IF serves as a simple-to-use tool to quickly assess the approximate position of the prosthesis. Previous studies have mainly focused on comparing the differences between different measurement methods based on postoperative pelvic AP X-ray and CT scans. Notably, investigations revealed nonequivalent results between anteversion based on IF measurements and postoperative measurements. The following reasons may be associated with the discrepancies in the measurements.

1. Pelvic tilt: Recent studies have reported a relationship between pelvic tilt and acetabular anteversion. Door et al. reported that pelvic tilt affected the surgeon's view of the acetabular side during surgery, with each 1° increase in anterior pelvic tilt resulting in a 0.7°– 0.8° decrease in acetabular anteversion^17^. Similar conclusions were drawn in a study on computerized navigation of the acetabular cup in which every 1° pelvic tilt resulted in a functional change of 0.7° in acetabular anteversion^18^. Additionally, a single-center study involving 59 patients demonstrated that the pelvic plane was not parallel to the horizontal plane during supine DAA-THA using a traction table^19^. Greater anteversion will be obtained if an acetabular cup is placed 15° from the horizontal plane on a significantly anteverted pelvis. Previous research has demonstrated that pelvic tilt leads to changes in acetabular anteversion, significantly affecting the intraoperative assessment of implant positioning^20–22^. Pelvic tilt alters the inclination and position of the pelvis, affecting its projected morphology in both the inlet and outlet views. For example, an anterior pelvic tilt can flatten the pelvic inlet and lead to changes in measurement parameters, such as pelvic diameter and angles in the pelvic inlet view. Our study found that the IF measurement of anteversion was 5.3° smaller than the postoperative measurement, which may be related to the presence of an anterior pelvic tilt during surgery.
2. Cup abduction inclination: Anterior and posterior pelvic rotation or rotation along the longitudinal axis of the body significantly affects cup anteversion, but have a lesser effect on the abduction angle^23^. Variations in cup abduction affect anteversion measurements, and a study developed a mathematical relationship to demonstrate this effect^24^. However, in this study, all abduction angles fell within the Lewinnek safety zone and had minimal effect on the anteversion measurement .
3. Central beam effect and X-ray offset: Due to the shorter source-to-film distance, the X-ray beam used in intraoperative C-arm imaging is more divergent than that used in postoperative radiographs, and the field of view obtained is smaller^25^. When taking pelvic or hip AP radiographs, the deviation of X-ray beams from the targeted hip center produces an offset angle that distorts the elliptical projection of the cup opening and makes the calculations inaccurate^26^. Similarly, moving the object away from the central X-ray beam causes variations in radiographic abduction and anteversion^24^. A study reported an average measurement error of 3.9° for radiographic anteversion when centering at a vertical or horizontal offset of 50 mm^27^. In the simplified calculation of anteversion by Widmer et al., the calculated angle needed to be increased by 5° to compensate for the effect of the X-ray offset, which was consistent with the correction factor of 5° or 6° used in this study^28^.
4. Parallax and C-arm Positions: The parallax effect is a potential source of error owing to the divergence of the X-ray beam, and its effect on radiographic anteversion measurements is generally in the range of 2°^29^. Furthermore, the C-arm primarily provides an inlet or outlet view, which may not be completely consistent with the postoperative standing AP view of the pelvis^30^. To obtain a larger visualization area, the position of the C-arm usually needs to be placed higher, potentially yielding a more "inlet" view of the pelvis, thereby reducing anteversion. Similarly, a change in the cephalad/caudal direction of the C-arm causes a change in anteversion^30^. Therefore, correct alignment of the X-ray beam and pubic symphysis using standardized fluoroscopic techniques may be an effective method to ensure measurement accuracy^31,32^. Additionally, ensuring to fix the center beam at the same height and position when obtaining postoperative AP pelvic radiographs or when using IF is necessary to avoid measurement errors caused by fluoroscopic imaging techniques.

Previous studies on the measurement of anteversion had recommended some calculation methods, including "Pradhan" method, "Lewinnek" method, "Widmer" method, "Liaw" method, "Hassan" method, and "Ackland" method. Among them, the "Lewinnek" method was the most widely used in measuring the anteversion and had excellent accuracy^8,27^. In this study, the PCC of the postoperative anteversion measured by the "Lewinnek" method and CT method was 0.959, which corresponded with the findings of Manjunath et al.^33^ Additionally, a strong correlation existed between IF and other measurement methods (PCC > 0.6). The intra- and inter-observer ICCs were all greater than 0.9, indicating that the three methods were reliable and repeatable. Furthermore, each point in Fig. 2B and C has a different degree of upward motion compared to that in Fig. 2A. This suggests that the anteversion obtained using the IF images may be underestimated.

Our study had some limitations. First, the different C-arm machines models used in IF may result in different correction factors. The C-arm used in this study was a 27-inch GE OEC ONE. Second, all surgeries in this study were performed by the same high-volume surgeon, and this correction factor may not apply to surgeons who have switched from a posterolateral approach to DAA. Third, although we matched the shape of the bilateral obturator with the preoperative shape during surgery, the position of the pelvis was not completely consistent with the preoperative position, which may have caused measurement bias to a certain extent. Fourth, the correction factor may vary depending on the position of the central beam of the C-arm relative to the central beam of the postoperative radiograph. Fifth, the sample size was small, and larger samples may be required for verification.

## Conclusion

Because of the combination of several factors, the IF assessment of cup anteversion in supine DAA-THA may lack complete accuracy. To obtain true cup anteversion intraoperatively, a correction factor of 5° or 6° may help adjust for the deviation; however, this requires further verification in large-sample experiments.

## Data Availability

The datasets used and analyzed during the current study are available from the corresponding author on reasonable request.
